# *Bothrops jararaca* snake venom: A reappraisal of its coagulant activity in humans, mice, and rats

**DOI:** 10.1371/journal.pntd.0014335

**Published:** 2026-05-26

**Authors:** Adrielly Viveiros Torres, Neusa Tadeu Penas Picon, Natacha Ferreira de Oliveira, Ana Teresa Azevedo Sachetto, Camila Martos Thomazini, Cynthia Zaccanini de Albuquerque, Vânia Gomes de Moura Mattaraia, Marcelo Larami Santoro

**Affiliations:** 1 Instituto Butantan, São Paulo, São Paulo, Brasil; 2 Faculdade de Medicina da Universidade de São Paulo, São Paulo, São Paulo, Brasil; Universidade Federal do Amazonas, BRAZIL

## Abstract

**Background:**

*Bothrops jararaca* snake venom (BjV) contains toxins that both activate and inhibit blood coagulation and platelets, leading to consumption coagulopathy, thrombocytopenia, endothelial dysfunction, and secondary fibrinolysis in human and animal victims of snakebite. Herein, we aimed to investigate the *in vitro* coagulant activity of BjV in human, mouse, and rat plasmas.

**Methodology/Principal findings:**

The coagulant activity of BjV was evaluated using normal human plasma and plasmas deficient in coagulation factors II, V, VII, VIII, IX, or X; in mice, normal plasma as well as plasma from *F8*^-/-^, *F9*^-/-^, *Vwf*^-/-^, and pearl (*Ap3b1*^-/-^) strains were analyzed, in addition to normal plasma from Wistar rats. The contributions of calcium ions, phospholipids, and blood cells to BjV-induced coagulant activity were assessed. Pharmacological inhibitors were used to investigate the generation of factor Xa and/or thrombin, and their roles in the coagulant activity of BjV. Statistical analyses were performed to compare minimum coagulant dose (MCD) values and their corresponding clotting curves. Human and rat plasmas showed greater sensitivity to the coagulant activity of BjV than mouse plasmas. Calcium ions and phospholipids significantly enhanced the coagulant activity of BjV. Remarkably, both red blood cells and platelets in whole blood markedly potentiated the coagulant activity of BjV. Pre-incubation of plasma samples with rivaroxaban and/or dabigatran demonstrated that the coagulant activity of BjV in rat plasmas primarily depended on prothrombin activation, whereas in human and mouse plasmas it involved both thrombin-like enzymes and prothrombin activators.

**Conclusions/Significance:**

This study reveals key differences in the coagulant activity of BjV in human and rodent plasmas, underscoring the need to considering these interspecies disparities when using animal models in comparative envenomation research.

## Author summary

Snakebites caused by *Bothrops jararaca*, popularly known as jararacas, are a major public health concern in South America, frequently leading to life-threatening blood clotting disorders. The venom of this species can trigger both excessive clotting and bleeding, but the precise mechanisms by which it interacts with human blood are still unclear. In this study, we examined how *B. jararaca* venom affects blood from humans, mice, and rats, which are commonly used in laboratory research. We found that the venom becomes much more active when components naturally found in blood—such as calcium, phospholipids, and especially blood cells like platelets—are present. This suggests that traditional methods using only plasma may underestimate venom activity. We also discovered that two blood clotting factors, prothrombin and factor V, play a central role in the venom action. Interestingly, we identified key differences among species: while mice and humans responded in a similar way to the venom, rats were much more sensitive to its procoagulant effects. These findings help explain why animal models vary in venom studies and highlight the importance of studying whole blood effects to better understand and treat snakebite-induced coagulopathy.

## Introduction

Snakebite envenomation is considered a neglected tropical disease by World Health Organization, with most cases occurring in Asia, Africa and Latin America. Approximately two million people are envenomed annually, and thousands still die as a result [1]. Viperidae snakes represent one of the most widespread groups of venomous reptiles worldwide. Their venoms comprise a diverse array of toxins that profoundly disrupt the delicate balance of hemostasis. Smaller animals such as rodents and amphibians, rapidly experience high circulating venom concentrations in the circulation, leading to thrombotic events, which culminate in early death. By contrast, in larger animals, the same absolute amount of injected venom is distributed within a greater blood volume and enters the circulation more gradually, resulting predominantly in hemorrhagic manifestations rather than acute thrombosis [2–5].

Snakebites are among the most frequently reported diseases in Brazil [6]. *Bothrops jararaca* snakes – present in southern Brazil, northern Argentina, and northeastern Paraguay [7] – are the main agents responsible for snakebites accidents in São Paulo State, Brazil [8]. Victims of *B. jararaca* snakebite envenomation typically manifest local effects at the site of the bite – including edema, ecchymoses, blisters, compartmental syndrome and necrosis –, as well as systemic signs of envenomation, such as spontaneous bleeding (gingival bleeding, hematuria and epistaxis) and blood incoagulability [9–12]. These hemostatic disturbances are caused by *B. jararaca* snake venom (BjV), which contain toxins that, during snakebite or experimental envenomation, disrupt blood coagulation and platelet function, leading to thrombocytopenia, endothelial injury, von Willebrand factor (VWF) dysfunction, and secondary fibrinolysis [9,13–24].

Although inherent differences exist among humans (*Homo sapiens*), mice (*Mus musculus*) and rats (*Rattus norvegicus*), laboratory mice and rats are widely accepted as valuable models for studying diverse physiological, pathological, pharmacological, and toxicological processes relevant to human health [25]. Laboratory animals, particularly mice, are also essential for quality control assays assessing the potency and safety of antivenoms used to treat snakebites [26]. In recent years, we have investigated how animal venoms, particularly BjV, trigger hemostatic disturbances in human victims and in experimental models. By using mouse, rats and human samples to study BjV-induced responses, we have observed both similarities and species-specific differences in hemostatic alterations. Following BjV inoculation, all three species exhibit fibrinogen consumption, thrombocytopenia, and local hemorrhage [18,27,28]. However, ecchymosis distant from the site of the bite and gingival bleeding –commonly observed in human patients [29] – are not observed in envenomed mice, rabbits and rats (personal observation). In addition, human and mouse platelets respond differently to BjV *in vitro* [30]. The coagulant activity of BjV on human plasma *in vitro* depends on both snake venom serine proteinases (SVSP) and snake venom metalloproteinases (SVMP) [31], although their relative contributions *in vivo* remain uncertain. On the other hand, SVMP are essential to induce coagulopathy in rats *in vivo* [27].

Patients bitten by *B. jararaca* snakes and rats experimentally injected with BjV exhibit marked consumption of plasma fibrinogen, factor V, and factor VIII, whereas prothrombin and factor X are only moderately consumed [19,22,32,33]. In addition to fibrinogen consumption, reduced levels of factor IX have been reported in snakebite envenomation, albeit to a lesser extent [32]. *Bothrops* snake venoms contain enzymes that cleave fibrinogen [34–37], as well as prothrombin and factors V, VIII, and X [38–42]. These venoms can also generate meizothrombin and thrombin *in vitro* [43] and *in vivo* [44]. Conversely, anticoagulant proteins have also been isolated and characterized [45–48], although they do not appear to play a major role in the coagulopathy evoked by envenomation [37]. While the biological activity of individual toxins has been evaluated *in vitro*, often in an isolated manner, few studies have integrated these effects or assessed their combined action in normal plasma, plasma deficient in specific coagulation factors, platelets, or whole blood.

Various methods have been employed to evaluate the coagulant activity of snake venoms *in vitro* [43,49–54]. Herein, we determined the minimum coagulant dose (MCD) – a widely used assay for evaluating the coagulant activity of snake venoms [55] – and analyzed clotting time curves to evaluate the contribution of different components to the coagulant activity of BjV. These analyses were carried out in blood samples from humans, and mice and rats, two experimental models commonly employed in studies of *Bothrops* venoms.

## Methods

### Ethics statement

All participants provided written consent to donate blood samples to this study. Procedures followed the Declaration of Helsinki and national regulations, and the study was approved by the National Human Research Ethics Committee (Plataforma Brasil, CAAE: 37958514.8.0000.0086, 52787521.0.0000.5469).

### Human blood samples (whole blood, platelet rich plasma, and platelet poor plasma)

Blood samples were obtained from healthy donors who reported no use of medication affecting hemostasis in the 10 days preceding collection. Venous blood was collected from the antecubital vein (9 volumes) and immediately transferred to tubes containing 3.2% trisodium citrate (1 volume). Samples were centrifuged at 1700 *g* for 15 min at room temperature to obtain platelet poor plasma (PPP). These samples were tested for prothrombin time (PT) and activated partial thromboplastin time (aPTT) before pooling; all samples were within the normal reference values. PPP samples were stored at -80°C. Platelet rich plasma (PRP, platelet counts: 270–373 × 10^9^/L) was obtained by centrifugation at 190 *g* for 15 min at room temperature, and used within 1 h of collection. Whole blood samples were anticoagulated in 3.2% sodium citrate, and also used within 1 h of collection; at the time of analysis, one volume of whole blood was diluted with one volume of sterile saline prewarmed at 37°C and transferred to cuvettes in the coagulometer. Plasmas deficient in coagulation factors II (prothrombin), V, VII, VIII, IX or X were obtained from Diagnostica Stago (France).

### Mouse and rat blood samples

Animal procedures were approved by the Animal Use Ethics Committee of the Instituto Butantan (CEUAIB protocols 4491070319, 8847060516, 5232120618 and 2181021015), and were in accordance with the National Guidelines from CONCEA [56], and ARRIVE guidelines [57]. The following mouse strains were used: Hemophilia A (***F8***^***-/-***^) (B6;129S-*F8*^*tm1Kaz*^/J) [58]; hemophilia B (***F9***^***-/-***^) (B6.129P2-*F9*^*tm1Dws*^/J) [59]; von Willebrand disease (***Vwf***^***-/-***^) (B6.129S2-*Vwf*^*tm1Wgr*^/J) [60]; pearl mice (***Ap3b1***^**pe**^) (B6Pin.C3-*Ap3b1*^*pe*^/J), a model of Hermansky-Pudlak syndrome with deficient platelet dense body content [61,62]; and **C57BL/6** mice (genetic background control strain), which is the genetic background for all mice used herein. *F8*^*-/-*^ and *F9*^*-/-*^ mice were obtained from CEMIB (Campinas, Brazil); *F8*^*-/-*^ mice were backcrossed to C57BL/6 mice for nine generations, using genotyping for selection. *Vwf*^*-/-*^ and *Ap3b1*^pe^ were obtained from The Jackson Laboratory (Bar Harbor, ME, USA); C57BL/6 are genetically monitored by CEMIB annually. Wistar Rats were obtained from the colony of Central Animal Facility, Instituto Butantan. All mice and rats were bred and maintained in a standardized conditions in the Central Animal Facility, Instituto Butantan, in barrier-controlled rooms with restricted access and controlled flow of personnel and materials. In addition, they were continuously protected by health status barriers (barrier autoclave, HEPA air filtration system, differential pressure, etc.). Animals were surveilled twice a year for the presence of pathogens and ectoparasites, according to FELASA recommendations [63]. Housing conditions included 22 ± 2°C room temperature, at 55 ± 10% humidity, 15–20 air changes per hour; polycarbonate microisolator cages (451-cm^2^ and 1154-cm^2^ floor area for mice and rats, respectively) with environmental enrichment (igloos, cotton, cardboard rolls, towel paper, disposable polypropylene cap for mice, and towel paper, disposable polypropylene cap and cotton for rats); autoclaved wood shavings and corncob xylan; free access to drinking water and irradiated Nuvilab CR1 pelleted feed (Nuvital1, Quimtia, Brazil); and weekly cage changes, and fresh autoclaved water twice weekly. The light cycle was 12 h light/ 12 h dark. The facility is accredited by the Conselho Nacional de Controle de Experimentação Animal (CONCEA).

Male mice (25–30 g) and male Wistar rats (250–300 g) were anesthetized with isoflurane (4% induction, and 2% maintenance). Whole blood (9 volumes) was collected via caudal vena cava puncture from mice using 1-mL plastic syringes fitted with 24G needles, and immediately transferred to tubes containing 3.2% trisodium citrate (1 volume). PPP was obtained by centrifugation at 2500 *g* for 15 min at room temperature and stored at −80°C. Individual PPP samples were tested for PT (DiaPlastin, DiaMed) before pooling; only samples within the established reference range were included. When anticoagulated whole blood samples were analyzed, they were diluted 1:1 with sterile saline prewarmed at 37°C immediately before use and transferred to cuvettes in the coagulometer.

Blood samples were collected from the abdominal aorta of male Wistar rat, using 10-mL plastic syringes fitted with 22G needles; blood (9 volumes) was immediately transferred to tubes containing 3.2% trisodium citrate (1 volume). PPP was obtained as described above, and only samples within the established reference range were included.

### Snake venom

Lyophilized crude venom from a pool of adult of *Bothrops jararaca* specimens (BjV) was kindly provided by the Laboratory of Herpetology, Instituto Butantan (Sistema Nacional de Gestão do Patrimônio Genético e do Conhecimento Tradicional Associado, SisGen AF375C2). Venom was stored at -20°C and weighed immediately prior to use in each experiment. Total protein concentration was 594.82 µg protein/mg crude venom [13]. BjV was two-fold serially diluted in either calcium-free Tyrode’s buffer or Tyrode’s buffer supplemented with calcium. The initial concentrations of BjV varied according to the substrate (PPP, PRP or whole blood) and species being tested; however, in general, stock solutions were prepared at 2.0 mg/mL and were two-fold serially diluted to 0.24 µg/mL. In some experiments, serial dilutions initiated at 8.0 mg/mL and extended to 1.9 ng/mL.

### Buffers

Calcium-free Tyrode’s buffer consisted of 137 mM NaCl, 2.7 mM KCl, 3**.**0 mM NaH_2_PO_4_, 10 mM HEPES, 5.6 mM dextrose, and 1 mM MgCl_2_ (pH 7.4). Tyrode’s buffer with calcium contained 137 mM NaCl, 2.7 mM KCl, 3.0 mM NaH_2_PO_4_, 10 mM HEPES, 5.6 mM dextrose, 1 mM MgCl_2_, and 2 mM CaCl_2_ (pH 7.4).

### Phospholipids

Bell and Alton platelet substitute (Diagen, BAPS04) was used as a source of phospholipids; 1 vial was diluted in 5 mL of Milli-Q water.

### Thrombin and factor Xa inhibitors

To evaluate the contribution of procoagulant BjV toxins to trigger the initiation of blood coagulation, two commercially available direct oral anticoagulants (DOACs) were employed, rivaroxaban, a direct factor Xa inhibitor, and dabigatran, a direct thrombin inhibitor. The active compounds were obtained by dissolving Xarelto tablets (40 mg, Bayer, Brazil), and Pradaxa capsules (110 mg, Boehringer, Brazil) in 1.0 and 2.75 mL of DMSO, respectively [64–68]. The tablets were triturated, the capsules were opened, both preparations were vortexed; the resulting suspensions were centrifuged at 10,000 *g* for 10 min, and the clear supernatants were collected and used as stock solutions. Rivaroxaban (40 mg/mL) and dabigatran (40 mg/mL) were added to PPP samples at a 1:100 dilution, resulting in final concentration of 200 µg/mL. At this concentration, rivaroxaban rendered PPP incoagulable in both PT and aPTT assays, whereas dabigatran prolonged clotting times by approximately fivefold relative to control samples. An equivalent volume of vehicle (DMSO) was added to control samples (S1 Fig).

### Inhibition of SVSP in mouse plasma

To evaluate the role of SVSP in the coagulant activity of BjV in C57BL/6 plasmas, BjV was serially diluted in Tyrode’s buffer with calcium, containing 8 mM 4-(2-aminoethyl)benzenesulfonyl fluoride hydrochloride (AEBSF, Sigma). Samples were incubated for 1 h at 37°C [69].

### Minimum coagulant dose (MCD)

The MCD – defined as the concentration of venom required to clot the plasma samples in 60 seconds [55] – was usually evaluated using two-fold serial dilutions of 2 mg/mL BjV (see above) in Tyrode’s buffer with calcium (in some experiments, calcium-free Tyrode’s buffer was used). Briefly, 100 µL of the substrate (PPP, PRP or whole blood) was transferred into a plastic cuvette containing a steel ball, and preheated for 120 s at 37°C. Thereafter, 25 µL of each BjV dilution was added, and the clotting was recorded up to 300 seconds [31]. All tests were performed in triplicates using a Start4 coagulometer (Stago, France) or a Coagmaster 2.0 coagulometer (Wama, Brazil). Clotting times were entered into Microsoft Excel spreadsheets, and following analyses in CurveExpert (version 1.4) for fitting models, curves for the clotting time (dependent variable) versus BjV concentration (independent variable) were generated in GraphPad Prism (version 8.0).

### Statistical analysis

Statistical analyses were performed using *Stata* (version 15, StataCorp, USA). Based on previous studies using CurveExpert and published data [31], a power-law function $\;(y=ax^{b})$ was identified as the most appropriate model for analyzing clotting time as a function of venom concentration. Both variables were log-transformed to satisfy assumptions of linearity and normality. Experimental groups were analyzed using regression models and inverse prediction techniques as detailed below. To assess whether the relationship between the independent and dependent variables differed across groups, a linear regression model with interaction terms was used, allowing testing whether the slopes and intercepts differed significantly between groups. Statistical significance of the interaction terms was evaluated to determine whether regression curves were distinct (see S1 Text). The predicted MCD value for each curve was calculated individually by inverse regression, using the estimated regression coefficients. To calculate confidence intervals, a nonparametric bootstrapping routine (5000 resamples) was implemented with the bootstrap command in Stata. Predicted MCD values were exponentiated (back transformation) to the original scale, and 95% confidence intervals (CI_95%_) were derived from the 2.5^th^ and 97.5^th^ percentiles of the bootstrap distribution. Pairwise group comparisons were considered statistically significant at α = 0.05 when 95% CIs did not overlap.

## Results

### The presence of calcium, phospholipids and blood cells in MCD assays enhance the coagulant activity of BjV

**Fig 1:**
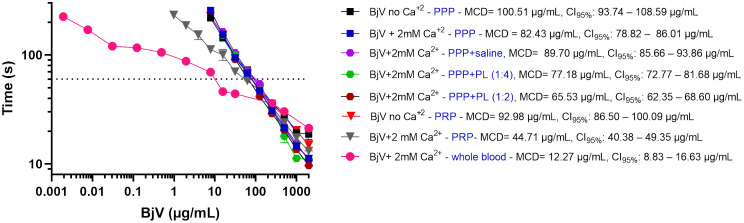
Minimum coagulant dose (MCD) curves of *Bothrops jararaca* venom (BjV) in citrated human plasma (PPP), platelet rich plasma (PRP) and whole blood. BjV was two-fold serially diluted, and clotting times were recorded in a coagulometer. The effects of calcium in the dilution solution (Tyrode’s buffer) of BjV, and the presence of phospholipids, platelets (PRP) and whole blood (containing all blood cells) were evaluated. Each point represents the mean ± s.e.m. from at least two replicates. MCD values and 95% confidence intervals (CI_95%_) were estimated using inverse regression with bootstrapping.

In the original description of the MCD assay [55], “physiological saline” was used as the diluent for snake venoms, but it not specified whether calcium was included. Since thrombin-like enzymes and procoagulant enzymes (i.e., prothrombin and factor X activators) present in BjV display enhanced activity in the presence of calcium ions [38,70], it was first investigated whether diluting BjV in Tyrode’s buffer containing calcium would affect MCD curves. Indeed, the presence of calcium in the dilution buffer of BjV potentiated the coagulant activity of BjV by approximately 20% (MCD = 82.43 µg/mL) compared with its absence (MCD = 100.51 µg/mL) (Fig 1). Such an increase in the coagulating activity of BjV has been already previously reported [50,71–73]. Thus, based on this observation, all subsequent experiments were carried out with BjV diluted in Tyrode’s buffer containing 2 mM CaCl_2_.

We next evaluated whether phospholipids, which physiologically facilitate the catalytic activity of coagulation factors, further enhanced the coagulant activity (Fig 1). One volume of plasma was incubated with one volume of saline (control) or increasing concentrations of the phospholipid suspension, reaching the dilutions of 1:4 and 1:2. As shown in Fig 1, clotting times was progressively shortened with phospholipid concentrations. This finding is consistent with previous reports demonstrating that calcium and phospholipids potentiate the procoagulant activity of *Bothrops* venoms [50,71–73]. Although BjV toxins cleave coagulation factors in the absence of phospholipids – unlike certain snake venom toxins, as group C and D prothrombin activators [74] –, their catalytic activity is significantly augmented in their presence.

Given that cellular membranes are the main physiological source of phospholipids for blood coagulation, we next examined whether blood cells enhance the coagulant activity of BjV (Fig 1). Firstly, we performed the coagulation tests in the presence of blood platelets (i.e., PRP). PRP markedly increased the coagulation activity of BjV (MCD = 44.71 µg/mL), surpassing the effect of purified phospholipids (MCD = 65.53 µg/mL). As previously reported [75–83], activated platelets provide a more effective procoagulant medium than phospholipid preparations, and also serves as a source of procoagulant mediators through platelet granule secretion, including polyphosphates, fibrinogen, platelet factor 4, and coagulation factors V and Va. Conversely, the omission of calcium reduced the coagulant activity of BjV, even in the presence of blood platelets, underscoring the synergistic role of calcium ions and cellular phospholipids. These findings indicate that blood platelets likely contribute to thrombin generation and to the overall coagulant activity of BjV *in vivo*, potentiating its effects. In addition, it should be noted that various C-type lectin-like proteins and SVSP present in BjV are capable of activating platelets, leading to phosphatidylserine externalization on the platelet surface, and the shedding of platelet-derived procoagulant microvesicles [14,17,30,84–86], which further amplify blood coagulation activation.

Finally, we investigated whether red blood cells, leukocytes, and platelets present in whole blood further accelerated the coagulant activity of BjV, as previously reported for other coagulant agents [87]. As shown in Fig 1, whole blood (diluted 1:2) markedly potentiated the coagulant activity of BjV (MCD = 12.27 µg/mL), representing an approximately 8-fold increase compared with plasma alone (MCD = 82.43 µg/mL). Coagulation time curves in whole blood also displayed distinct slopes compared with plasma, indicating that all blood cells accelerate coagulant activity of BjV *in vivo*. To our knowledge, this is the first study to demonstrate that blood cells enhance the coagulant activity of BjV.

### Prothrombin and factor V are the major contributors to the coagulating activity of BjV *in vitro*

**Fig 2:**
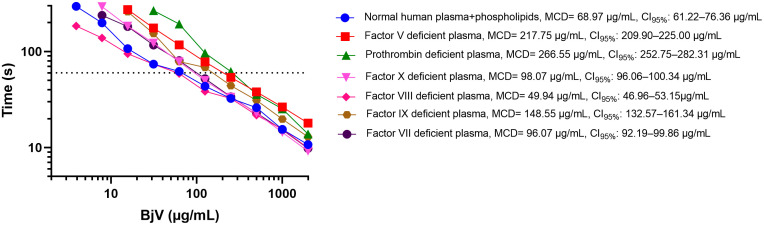
Minimum coagulant dose (MCD) curves of *Bothrops jararaca* venom (BjV) in citrated normal and deficient human plasmas (PPP) in the presence of phospholipids. BjV was two-fold serially diluted in Tyrode’s buffer containing 2 mM CaCl_2_. Clotting times were recorded in a coagulometer. Each point represents the mean ± s.e.m. from at least two replicates. MCD values and 95% confidence intervals (CI_95%_) were estimated using inverse regression with bootstrapping.

Since coagulation tests rely on fibrin polymerization, fibrinogen is the only blood coagulation factor whose absence cannot be tested in samples. To identify which blood coagulation factors, besides fibrinogen, were involved in the coagulant activity of BjV in human plasma, we tested commercial plasmas deficient in coagulation factors II, V, VII, VIII, IX, or X, in the presence of 2 mM CaCl_2_ and phospholipids (Fig 2). Elevated MCD values and statistical comparisons analyses revealed that BjV depended primarily on prothrombin and factor V. Besides thrombin-like enzymes, these results are consistent with findings showing that prothrombin activators are major blood coagulation activators in BjV [13]. Factors VII, IX and X also contributed, but to a lesser extent. Interestingly, plasma deficient in factor VIII displayed even lower MCD values than normal plasma, suggesting that factor VIII, even though it is activated by thrombin, is not as important as factor IX to the coagulant activity of BjV. These results were also replicated in mouse plasma (see underneath). However, when a single concentration of BjV that induced clotting within approximately 18 seconds was used, previous studies reported no detectable differences in the coagulant activity of BjV in plasmas deficient in factor VIII or IX [71,72].

In human snakebite patients, the main blood coagulation factors consumed are fibrinogen and factors V and VIII, while levels of factors X and IX are generally preserved; prothrombin levels, however, vary markedly [19,32]. Similarly, in rats and mice injected s.c. with BjV, we observed steady decreases in fibrinogen, factor V and VIII, whereas factor X levels fell to around 40% of those of controls, and prothrombin levels had mild reductions [27,33]. However, in dogs injected i.v. with BjV, levels of fibrinogen, prothrombin and factor X declined steadily [15]. These differences likely reflect both the diverse routes of venom administration and species-specific differences of BjV in mammal blood [13,31].

Thrombocytin, a SVSP from *Bothrops atrox*, has been reported to directly activate factor V [41], and, in a recent paper, the prothrombin-activator of *B. atrox* has been shown to depend on factor Va for its activity [88]. Considering the dependence of the coagulant activity of BjV on factor V *in vitro*, and the pronounced consumption of this factor V during snakebites, it is plausible that BjV also contains factor V activators, a hypothesis that warrants further investigation. Supporting this idea, meizothrombin – the major product of prothrombin cleavage by most *Bothrops* prothrombin activators [38,39,89,90], except *B. moojeni* [91] – is a poor activator of factor V compared to α-thrombin [92]. Thus, meizothrombin alone cannot account for factor V activation observed *in vitro* and *in vivo.*

### Comparative coagulant activity of BjV in mouse and rat plasmas

**Fig 3:**
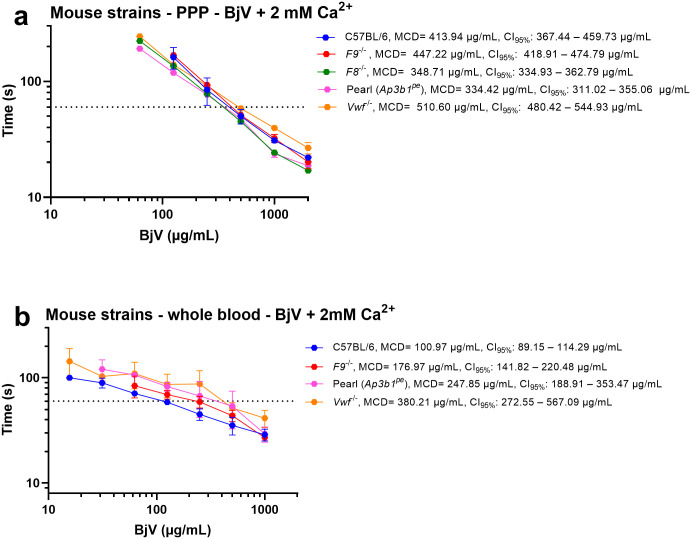
Minimum coagulant dose (MCD) curves of *Bothrops jararaca* venom (BjV) in citrated mouse platelet poor plasma (PPP) (a) or whole blood (b). BjV was two-fold serially diluted, and clotting times were recorded using a coagulometer. The effects of diverse hemostatic defects in mouse strains were analyzed. Each point represents the mean ± s.e.m. from at least two replicates. MCD values and 95% confidence intervals (CI_95%_) were estimated using inverse regression with bootstrapping.

A previous study demonstrated that BjV exhibits distinct coagulant activity in rabbit versus human plasmas and fibrinogen [31]. Herein we extend these findings to mouse and rat plasmas. Knockout mice are frequently employed as models to study normal and abnormal hemostasis [93–96]. In order to minimize variability among strains [97], we used mice on the same genetic background of *F8*^*-/-*^, *F9*^*-/-*^, *Vwf*^*-/-*^ and *Ap3b1*^pe^ mice, i.e., C57BL/6 mice. Compared with humans, mouse plasma and whole blood displayed higher MCD values. This resistance to the coagulant activity of BjV (Fig 3) may reflect the presence of natural inhibitors such as murinoglobulin [98,99], consistent with coevolution between snakes and mice, which are natural preys of snakes. By contrast, rabbits, which are non-natural preys for South American snakes—are highly sensitive to the procoagulant activity of BjV, even though their fibrinogen molecule is resistant to the clotting activity of thrombin-like enzymes [31,34]. Additionally, interspecies structural differences in coagulation factors [100,101], and variations in their plasma levels likely contribute to these divergent responses [100]. Despite these differences, several similarities emerged between humans and mice. First, as in humans (Fig 2), factor IX deficiency prolonged BjV-induced clotting in PPP, whereas factor VIII deficiency shortened clotting times compared with C57BL/6 controls (Fig 3). Thus, both in humans and mice, factor IX appears to be important for BjV-induced coagulation *in vitro*, while factor VIII deficiency paradoxically enhances its coagulant activity. Interestingly, in human snakebites, factor IX is not significantly consumed, whereas factor VIII is markedly reduced [19,22,32]. The mechanisms underlying these discrepancies remain to be clarified. Second, as in humans, mouse whole blood (Fig 3b) was more sensitive to the coagulant activity of BjV than PPP (Fig 3a). It is intriguing that *Vwf*^-/-^ mice, lacking VWF – a glycoprotein that does not participate directly in the blood coagulation cascade, but is a carrier for circulating factor VIII [102] –, were the most sensitive strain to BjV in both PPP and whole blood (Fig 3a and 3b). Given that VWF is a direct target of BjV [22], the VWF cleavage may impair factor VIII stability and availability, thereby contributing to factor VIII depletion during envenomation. At this stage, it would be highly speculative to propose a mechanism whereby factor VIII depletion in plasma enhances the coagulant activity of BjV in mice and humans. Future investigations should examine the activation and proteolytic processing of coagulation factors V and VIII by BjV, under both *in vivo* and *in vitro* conditions, as well as assess the biological consequences of this interaction.

Platelets from *Ap3b1*^*pe*^ mice contain defective platelets on phosphatidylserine exposure, and whose dense bodies are deficient in storage of adenine nucleotides and serotonin, rendering them defective on secretion-dependent procoagulant activity [30,103]. PPP from *Ap3b1*^*pe*^ mice was more sensitive to the coagulant activity of BjV than that of C57BL/6, whereas their whole blood, containing disordered platelets, was more unresponsive. These findings indicated that platelet activation is an important mechanism whereby BjV promotes activation of the coagulation cascade. In line with this, patients with Hermansky-Pudlak syndrome caused by AP3b1 deficiency exhibit normal plasma coagulation, but impaired platelet aggregation, similar to pearl mice [104].

### The use of inhibitors to study the coagulant activity of BjV

**Fig 4:**
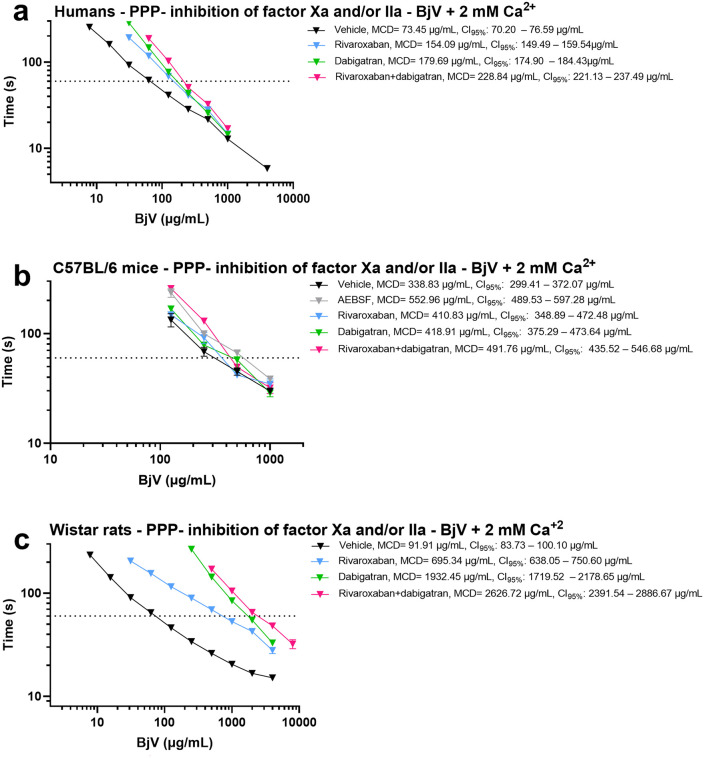
Minimum coagulant dose (MCD) curves of *Bothrops jararaca* venom (BjV) in citrated platelet poor plasmas (PPP) from humans (a), C57BL/6 mice (b), and Wistar rats (c). BjV was two-fold serially diluted, and clotting times were recorded using a coagulometer. The effects of rivaroxaban (200 µg/mL, final concentration), a factor-Xa inhibitor and/or dabigatran (40 µg/mL, final concentration), a direct thrombin inhibitor, were evaluated. DMSO was used as a vehicle to solubilize rivaroxaban and dabigatran. Each point represents the mean ± s.e.m. from at least two replicates. MCD values and 95% confidence intervals (CI_95%_) were estimated using inverse regression with bootstrapping.

We next addressed whether (meizo)thrombin generation and factor X activation contribute to the coagulant activity of BjV, and whether these mechanisms differ among rats, mice and humans. For this purpose, we employed rivaroxaban, a potent factor Xa inhibitor, and dabigatran, a direct thrombin inhibitor, two novel oral anticoagulants currently in the clinical management of thromboembolism [105]. When comparing the MCD in vehicle-treated plasma (controls) (Fig 4), BjV exhibited the highest coagulant activity in human plasma, followed in decreasing order by rat and mouse plasma. When dabigatran and/or rivaroxaban are used, it is clear that the participation of thrombin, factor Xa and thrombin-like enzymes are different among species.

In human plasma (Fig 4a), (meizo)thrombin generation appeared slightly more relevant than factor Xa generation, but both contributed substantially to the coagulant activity of BjV. Combined inhibition with dabigatran and rivaroxaban only partially reduced BjV activity, indicating that residual thrombin-like enzymes still sustain coagulation. These results are consistent with prior studies using inhibitors of snake venom metalloproteinases (SVMP; factor X and prothrombin activators) and snake venom serine proteinases (SVSP; thrombin-like enzymes) [31].

Likewise in human plasma, in C57BL/6 mouse plasma (Fig 4b), rivaroxaban and dabigatran exerted comparable inhibitory effects. When both inhibitors were used together, the residual thrombin-like activity of BjV – i.e., the remaining coagulant activity after inhibition of (meizo)thrombin generation and factor Xa – yielded MCD values similar to those obtained after pretreatment of BjV with AEBSF, a SVSP inhibitor. These findings indicate that, in both mice and humans, BjV activates coagulation by means of thrombin-like enzymes and procoagulant activators with comparable relevance. Thus, mice represent a suitable experimental model for studying BjV-induced coagulopathy.

In contrast, Wistar rat plasma (Fig 4c) exhibited a distinct inhibitory profile. Based on MCD values and clotting curve patterns, factor X and prothrombin activators had a more pronounced role in rat plasma than in human or mouse plasma. Importantly, (meizo)thrombin generation emerged as the predominant determinant of BjV coagulant activity in rats. When both inhibitors were combined, only minimal residual activity remained, indicating that thrombin-like enzymes contribute little to coagulation in this species, in agreement with previous reports [27,33]. Despite their close taxonomic relationship, rats and mice diverge markedly in their hemostatic response to BjV, both *in vitro* and *in vivo*.

## Discussion

Our study addressed questions that are rarely investigated in studies of snake venom coagulant activity – such as the participation phospholipids, blood cells, coagulation factors, and interspecies differences among experimental models. We demonstrated that: (a) the presence of calcium ions in the dilution buffer of BjV and phospholipids in the reaction mixture enhances the coagulant activity of BjV in MCD assays, confirming previous findings; (b) prothrombin and factor V make a decisive contribution to the coagulant activity of BjV; (c) the absence of factor VIII was associated with an increase in the coagulant activity of BjV; (d) platelets and whole blood further potentiate the coagulant activity of BjV, thereby closely mimicking the physiological conditions encountered during envenomation; (e) thrombin and factor Xa generation differentially contributes to the coagulant activity of BjV in mice, rats and humans.

Although this study provides novel data, we acknowledge several limitations. First, baseline interspecies differences in coagulation parameters may contribute to variability in MCD values, and represent an uncontrolled source of heterogeneity. While direct normalization across species is challenging due to fundamental physiological differences, the observed differences do reflect the interaction between venom activity and species-specific hemostatic backgrounds, rather than venom effects in isolation. Second, the data presented herein address exclusively the *in vitro* contribution of BjV toxins to blood coagulation activation in humans, mice and rats, as well as the involvement of specific coagulation factors and blood cells. Whether these components exert similar roles *in vivo* was not investigated. As recently reported, not all effects of snake venom toxins *in vitro* – particularly anticoagulant activities – are necessarily reproduced *in vivo* [106]. In future studies, we intend to investigate animal models deficient in coagulation factors and with platelet functional abnormalities to better delineate their *in vivo* contribution to the hemostatic disturbances induced by snakebite envenomation. Nonetheless, it should be recognized that experimental models do not always reliably predict the pathophysiological processes occurring in humans. Third, this study relied on commercial formulations of dabigatran (Pradaxa) and rivaroxaban (Xarelto), which contain multiple excipients in their pharmaceutical formulations. Although precautions were taken to remove insoluble components during drug preparation, the potential influence of soluble excipients on the coagulation assays could not be excluded. Even so, our results suggest that inhibition of (meizo)thrombin activity by DOACs, such as dabigatran, may attenuate excessive blood coagulation activation following envenomation by snakes whose venoms contain prothrombin activators, including *B. jararaca* snakes. In fact, DOACs are clinically easier to manage than warfarin or heparin, and are associated with fewer adverse effects [107]. However, the clinical relevance and safety of modulating blood coagulation activation by DOACs during snakebite envenomation require careful evaluation in future studies. Fourth, only a single snake venom was examined in this study, and different results might have been obtained if venoms from other snake species were evaluated. Moreover, even venoms obtained from the same species may vary according to ontogenetic stage [13], sex [108], geographical origin [109] or intraspecific variation [110],potentially leading to distinct effects on blood coagulation.

The Lee-White and the 20-minute whole-blood clotting time have long been used worldwide as an inexpensive, bedside methods to diagnosis coagulopathy in snakebite victims. However, they have been criticized for their limited sensitivity and lack of standardization [111,112]. Research is required to develop more reliable and cheap clotting tests to be used for the diagnosis and treatment of consumptive coagulopathy, and rapid tests may be a future alternative [113,114]. Although whole-blood clotting tests are regarded as outdated compared with more standardized and controlled assays performed in clinical laboratories [115–118], our findings provide renewed relevance to their use. Specifically, BjV exhibited a higher coagulant activity in whole blood than in PPP, thereby more closely mimicking the physiological conditions present in the circulation of envenomed patients. Consequently, existing diagnostic approaches for evaluating coagulopathy in snakebite envenomation should be contextualized and interpreted in light of these observations, which may contribute to improving antivenom administration and overall clinical management.

We believe that our findings are relevant both to clinicians managing patients envenomed by *B. jararaca* as to researchers and the technical staff who use rodent models for the characterization of venom biological activities and the production of antivenoms. As demonstrated herein, the scenario of comparing the same snake venom across different species, even among mammals, is substantially complex. By focusing solely on the coagulant activity of BjV, we identified marked interspecies differences among humans, mice and rats. Snake venom toxins that are critical to the pathophysiology of envenomation in one species may be less relevant in another. For example, toxins that induce produce fibrinogen consumption in equines – which are likely to be highly immunogenic in this species – may play a limited role in rodents or even in humans. Our findings reinforce previous findings stating that such species-specific differences in venom activity should be carefully considered when evaluating antivenom potency and when translating experimental findings from animal models to the clinical regimen of antivenom used in humans [119,120].

Although whole blood is infrequently used to evaluate the coagulant activity of snake venoms *in vitro*, apart from thromboelastographic approaches [53,121,122], our findings indicate that the whole blood clotting time remains a clinically meaningful parameter in envenomed patients. This assay is inexpensive, rapid and easily performed at the bedside, and it more closely reflects physiological conditions, as both platelets and red blood cells significantly potentiate the procoagulant effects of BjV in circulation. From a public health perspective, this is particularly relevant for low- and middle-income regions where snakebite is endemic and access to sophisticated laboratory assays is limited. Our results therefore support the broader use of whole blood in venom research, extending beyond hemostasis-related investigations [123]. Historically, major advances in the understanding of hemostasis have arisen from studies of snake venom toxins that target platelet receptors (e.g., glycoprotein Ib and VWF [124], glycoprotein VI [125], and CLEC-2 [126]) – as well as coagulation factors [127–129]. The present findings reinforce the notion that snake venoms remain an invaluable resource for uncovering novel mechanisms in hemostasis.

## Supporting information

S1 Fig Effects of increasing concentrations (1 µL) of rivaroxaban (a direct factor Xa inhibitor) or dabigatran (a direct thrombin inhibitor) spiked in human citrated plasma (100 µL) on coagulation tests used clinically for evaluation of the blood coagulation extrinsic and common pathways (prothrombin time, PT) and intrinsic and common pathways (activated partial thromboplastin time, aPTT). DMSO was used as vehicle for dissolving rivaroxaban and dabigatran, and served as the control. Results are expressed as duplicate measurements for each dilution. Tables reporting the clotting times for dabigatran and rivaroxaban in PT and aPTT assays are provided in the Supplementary Information.
(PDF)

S1 Text List of statistical analyses for Figs 1–4 in the main text. (PDF)

S1 Table Raw clotting time data used to generate the dose–response curves presented in Figs 1–4 of the main text. Each worksheet corresponds to a specific experimental condition and includes individual clotting time measurements plotted as a function of *Bothrops jararaca* venom concentration.
(XLSX)
