# Supplementary figures and images for: Bothrops jararaca snake venom: A reappraisal of its coagulant activity in humans, mice, and rats
Source: PLoS Negl Trop Dis. 2026 May 26;20(5):e0014335. doi: 10.1371/journal.pntd.0014335 (PMC13210399; doi:10.1371/journal.pntd.0014335)

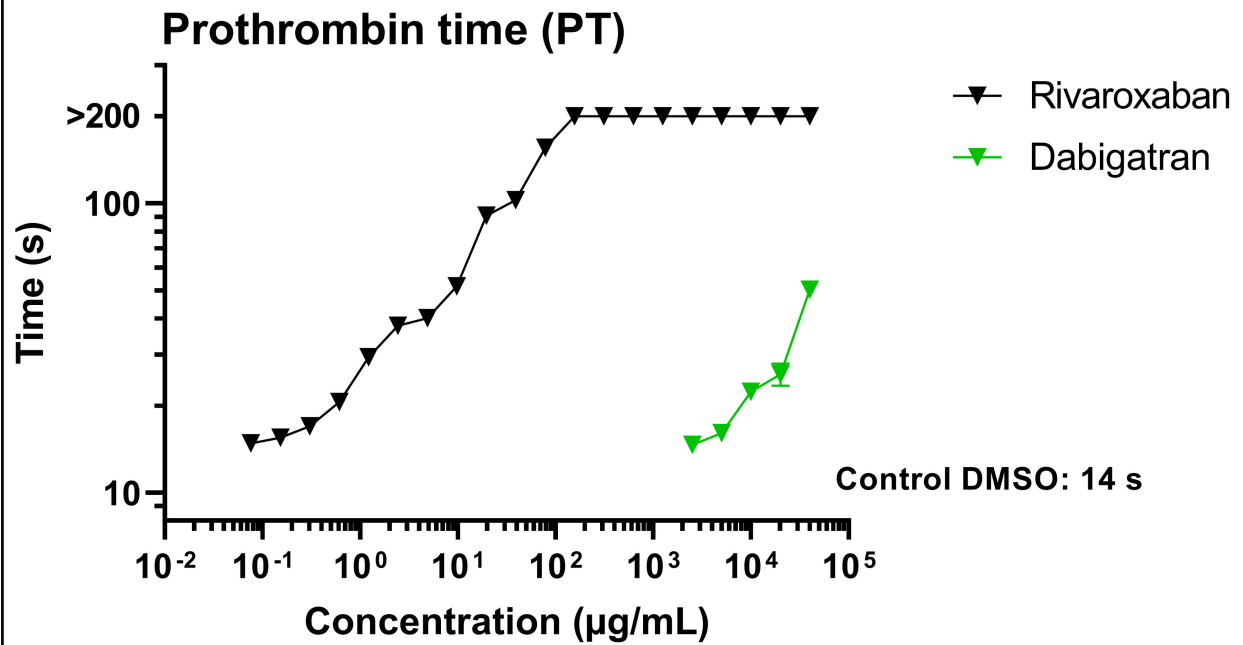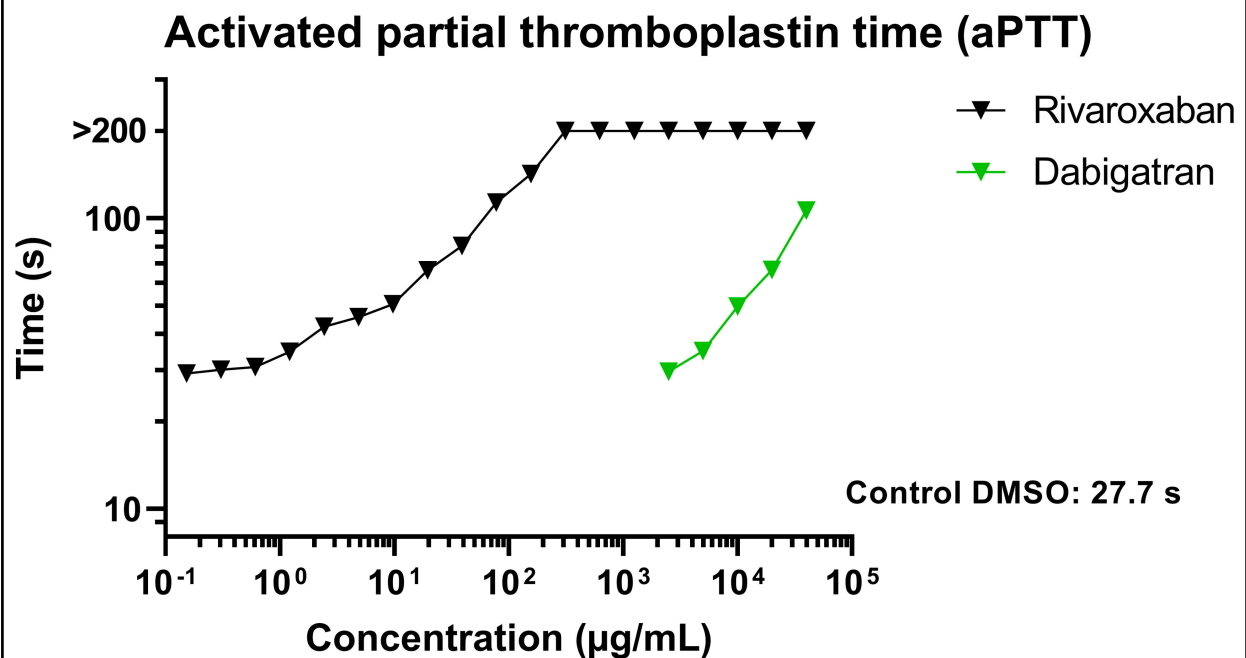

Figure1.tif

Supplement: S1 Fig — DMSO was used as vehicle for dissolving rivaroxaban and dabigatran, and served as the control. Results are expressed as duplicate measurements for each dilution. Tables reporting the clotting times for dabigatran and rivaroxaban in PT and aPTT assays are provided in the Supplementary Information. (PDF) [file pntd.0014335.s001.pdf]
