# Supplementary material for: Bothrops jararaca snake venom: A reappraisal of its coagulant activity in humans, mice, and rats
Source: PLoS Negl Trop Dis. 2026 May 26;20(5):e0014335. doi: 10.1371/journal.pntd.0014335 (PMC13210399; doi:10.1371/journal.pntd.0014335)
Supplement: S1 Text — (PDF) [file pntd.0014335.s002.pdf]

Bothrops jararaca snake venom: a reappraisal of its coagulant activity in humans, mice, and rats

Adrielly Viveiros Torres[1,2], Neusa Tadeu Penas Picon[1], Natacha Ferreira de Oliveira[1,2], Ana Teresa Azevedo Sachetto[1,2], Camila Martos Thomazini[1,2], Cynthia Zaccanini de Albuquerque[1], Vânia Gomes de Moura Mattaraia[1], Marcelo Larami Santoro[1,2,*]

1. Instituto Butantan, São Paulo - SP, Brasil.
2. Faculdade de Medicina da Universidade de São Paulo, São Paulo -SP, Brasil.

Statistical analyses for Figures 1-4

Figure 1 (**main text**) -Statistical analyses were performed in Stata 15.0. Only statistically significant differences are shown below.

Statistical differences between (humans):
PPP- MCD BjV no calcium + saline versus MCD BjV no calcium + phospholipids (1:2), p<0.05
PPP - MCD BjV no calcium + saline versus MCD BjV + calcium + saline, p<0.05
PPP - MCD BjV no calcium + saline versus MCD BjV + calcium + phospholipids (1:2), p<0.05
PPP - MCD BjV no calcium + saline versus MCD BjV + calcium + phospholipids (1:4), p<0.05
PPP - MCD BjV no calcium + phospholipids (1:2) versus MCD BjV + calcium + saline, p<0.05
PPP - MCD BjV no calcium + phospholipids (1:2) versus MCD BjV + calcium + phospholipids (1:2), p<0.05
PPP - MCD BjV no calcium + phospholipids (1:2) versus MCD BjV + calcium + phospholipids (1:4), p<0.05
PPP - MCD BjV + calcium + saline versus MCD BjV + calcium + phospholipids (1:2), p<0.05
PPP - MCD BjV + calcium + saline versus MCD BjV + calcium + phospholipids (1:4), p<0.05
PPP - MCD BjV + calcium + phospholipids (1:2) versus MCD BjV + calcium + phospholipids (1:4), p<0.05

MCD BjV no calcium+ PPP/saline versus MCD BjV + calcium + plasma/saline, p<0.05
MCD BjV no calcium+ PRP/saline versus MCD BjV + calcium + PRP/saline, p<0.05
MCD BjV + calcium + PPP/saline versus MCD BjV + calcium + PRP/saline, p<0.05
MCD BjV + calcium + PPP/saline versus MCD BjV + calcium + whole blood/saline, p<0.05
MCD BjV + calcium + PRP/saline versus MCD BjV + calcium + whole blood/saline, p<0.05

Slope BjV no calcium+ PPP/saline versus slope BjV + calcium + plasma/saline, p< 0.001
Slope BjV no calcium+ PPP/saline versus slope BjV + calcium + PRP/saline, p< 0.001
Slope BjV no calcium+ PPP/saline versus slope BjV + calcium + whole blood/saline, p< 0.001
Slope BjV + calcium + PPP/saline versus slope BjV + calcium + PRP/saline, p<0.001
Slope BjV + calcium + PPP/saline versus slope BjV + calcium + whole blood/saline, p<0.001

Figure 2 (main text) - Statistical analyses were performed in Stata 15.0. Only statistically significant differences are shown below.

Statistical differences between (humans):
MCD Normal PPP + calcium + phospholipids versus MCD normal PPP + calcium, $p<0.05$
MCD prothrombin deficient PPP + calcium versus MCD prothrombin deficient PPP + calcium + phospholipids, $p<0.05$
MCD factor X deficient factor X deficient PPP + calcium versus MCD factor X deficient PPP + calcium + phospholipids, $p<0.05$
MCD factor VIII deficient PPP + calcium versus MCD factor VIII deficient PPP + calcium + phospholipids, $p<0.05$
MCD Normal PPP +calcium + phospholipids versus MCD factor V deficient PPP + calcium + phospholipids, $p<0.05$
MCD Normal PPP + calcium + phospholipids versus MCD prothrombin deficient PPP + calcium + phospholipids, $p<0.05$
MCD Normal PPP + calcium + phospholipids versus MCD factor X deficient PPP + calcium + phospholipids, $p<0.05$
MCD Normal PPP + calcium + phospholipids versus MCD factor VIII deficient PPP + calcium + phospholipids, $p<0.05$
MCD Normal PPP + calcium + phospholipids versus MCD factor IX deficient PPP + calcium + phospholipids, $p<0.05$
MCD Normal PPP + calcium + phospholipids versus MCD factor VII deficient PPP + calcium + phospholipids, $p<0.05$

Slope normal PPP + calcium versus slope normal PPP + calcium + phospholipids, $p< 0.001$
Slope normal PPP + calcium versus slope prothrombin deficient PPP + calcium, $p< 0.001$
Slope normal PPP + calcium versus slope factor VIII deficient PPP + calcium, $p<0.027$
Slope normal PPP + calcium versus slope factor IX deficient PPP + calcium, $p=0.005$
Slope normal PPP + calcium + phospholipids versus slope factor V deficient PPP + calcium + phospholipids, $p=0.001$
Slope normal PPP + calcium + phospholipids versus slope prothrombin deficient PPP +calcium + phospholipids, $p<0.001$
Slope normal PPP + calcium + phospholipids versus slope $F10^{-/-}$ PPP + calcium + phospholipids, $p=0.593$
Slope normal PPP + calcium + phospholipids versus slope factor VIII deficient PPP + calcium + phospholipids, $p<0.001$
Slope normal PPP + calcium + phospholipids versus slope factor IX deficient PPP + calcium + phospholipids, $p=0.098$
Slope normal PPP + calcium + phospholipids versus slope factor IX deficient PPP + calcium + phospholipids, $p=0.036$
Slope factor V deficient PPP + calcium versus slope factor V deficient PPP + calcium + phospholipids, $p=0.285$
Slope prothrombin deficient PPP + calcium versus slope prothrombin deficient PPP + calcium + phospholipids, $p<0.001$
Slope factor X deficient PPP + calcium versus slope factor X deficient PPP + calcium + phospholipids, $p=0.008$
Slope factor VIII deficient PPP + calcium versus slope factor VIII deficient PPP + calcium + phospholipids, $p<0.001$
Slope factor IX deficient PPP + calcium versus slope factor IX deficient PPP + calcium + phospholipids, $p=0.004$
Slope factor VII deficient PPP + calcium versus slope factor VII deficient PPP + calcium + phospholipids, $p=0.046$

Figure 3 (main text) - Statistical analyses were performed in Stata 15.0. Only statistically significant differences are shown below.

a) **PPP**

Statistical differences between:
MCD C57BL6 versus *F8*$^{-/-}$, p<0.05
MCD C57BL6 versus Pearl, p<0.05
MCD C57BL6 versus *Vwf*$^{-/-}$, p<0.05
MCD *F8*$^{-/-}$ versus *F9*$^{-/-}$, p<0.05
MCD *F8*$^{-/-}$ versus *Vwf*$^{-/-}$, p<0.05
MCD *F9*$^{-/-}$ versus Pearl, p<0.05
MCD *F9*$^{-/-}$ PPP versus *Vwf*$^{-/-}$, p<0.05
MCD Pearl versus *Vwf*$^{-/-}$, p<0.05

Slope C57BL6 versus slope *Vwf*$^{-/-}$, p<0.021
Slope *F8*$^{-/-}$ versus slope *Vwf*$^{-/-}$, p= 0.001
Slope *F9*$^{-/-}$ versus slope Pearl, p= 0.025
Slope *F9*$^{-/-}$ versus slope *Vwf*$^{-/-}$, p= 0.001
Slope Pearl versus slope *Vwf*$^{-/-}$, p= 0.001

b) **Whole blood**

Statistical differences between:

MCD C57BL6 versus MCD *F9*$^{-/-}$, p<0.05
MCD C57BL6 versus MCD Pearl, p<0.05
MCD C57BL6 versus MCD *Vwf*$^{-/-}$, p<0.05
MCD *F9*$^{-/-}$ versus *Vwf*$^{-/-}$, p<0.05

Slope C57BL6 versus slope Pearl, p=0.011

Figure 4 (main text) - Statistical analyses were performed in Stata 15.0. Only statistically significant differences are shown below.

Statistical differences between:

a) **PPP- humans**

MCD vehicle (DMSO) versus dabigatran, $p<0.05$
MCD vehicle (DMSO) versus rivaroxaban, $p<0.05$
MCD vehicle (DMSO) versus dabigatran + rivaroxaban, $p<0.05$
MCD dabigatran versus rivaroxaban, $p>0.05$
MCD dabigatran versus dabigatran + rivaroxaban, $p<0.05$
MCD rivaroxaban versus dabigatran + rivaroxaban, $p<0.05$

Slope vehicle (DMSO) versus dabigatran, $p<0.001$
Slope vehicle (DMSO) versus rivaroxaban, $p<0.001$
Slope vehicle (DMSO) versus dabigatran + rivaroxaban, $p<0.001$
Slope dabigatran versus rivaroxaban, $p<0.001$
Slope dabigatran versus dabigatran + rivaroxaban, $p<0.001$
Slope rivaroxaban versus dabigatran + rivaroxaban, $p<0.001$

b) **PPP – mice**

MCD vehicle (DMSO) versus AEBSF, $p<0.05$
MCD vehicle (DMSO) versus dabigatran, $p<0.05$
MCD vehicle (DMSO) versus rivaroxaban + dabigatran, $p<0.05$
MCD AEBSF versus rivaroxaban, $p<0.05$
MCD AEBSF versus dabigatran, $p<0.05$

Slope BjV versus rivaroxaban + dabigatran, $p< 0.001$
Slope AEBSF versus rivaroxaban + dabigatran, $p= 0.001$
Slope rivaroxaban versus rivaroxaban + dabigatran, $p= 0.008$
Slope dabigatran versus rivaroxaban + dabigatran, $p= 0.002$

c) **PPP - Wistar rats**

MCD vehicle (DMSO) versus dabigatran, $p<0.05$
MCD vehicle (DMSO) versus rivaroxaban, $p<0.05$
MCD vehicle (DMSO) versus dabigatran+ rivaroxaban, $p<0.05$
MCD dabigatran versus rivaroxaban, $p<0.05$
MCD dabigatran versus rivaroxaban, $p<0.05$
MCD dabigatran versus dabigatran + rivaroxaban, $p<0.05$
MCD rivaroxaban versus dabigatran + rivaroxaban, $p<0.05$

Slope vehicle (DMSO) versus dabigatran, $p<0.001$
Slope vehicle (DMSO) versus rivaroxaban, $p<0.001$
Slope vehicle (DMSO) versus dabigatran + rivaroxaban, $p<0.001$
Slope dabigatran versus rivaroxaban, $p<0.001$
Slope dabigatran versus dabigatran + rivaroxaban, $p<0.001$
Slope rivaroxaban versus dabigatran + rivaroxaban, $p=0.002$
